# Giant paravertebral schwannoma near the lumbar nerve roots with bone destruction: A case report

**DOI:** 10.1097/MD.0000000000017341

**Published:** 2019-10-18

**Authors:** Hangjun Chen, Qiang Xu, Ping Zhan, Yuan Liu, Min Dai, Bin Zhang

**Affiliations:** Department of Orthopedics, Artificial Joints Engineering and Technology Research Center of Jiangxi Province, The First Affiliated Hospital of Nanchang University, Nanchang, Jiangxi, China.

**Keywords:** giant schwannoma, lumbar vertebra, misdiagnosis, surgery, treatment

## Abstract

**Rationale:**

Schwannomas grow slowly, originating from the Schwann cells of the nerve sheath. Schwannomas of cranial origin have the highest incidence, followed by intraspinal schwannomas. However, paravertebral schwannoma is rare, and to our knowledge, giant paravertebral schwannomas near the lumbar nerve roots with bone destruction are extremely rare.

**Patient concerns:**

A 47-year-old Chinese woman complained of lower back soreness and a sensation of a bulging lumbar disc with no obvious cause for the past 3 years.

**Diagnosis:**

Lumbar magnetic resonance imaging showed a large mass with uneven density, 17 × 12 × 15 cm in size, located to the right of the 4th lumbar with obvious bony destruction. Histopathology and immunohistochemistry confirmed that this mass was a benign schwannoma.

**Interventions:**

Complete resection of the tumor (measuring about 17 × 12 × 15 cm in size) and vertebral reconstruction using internal fixation were performed.

**Outcomes:**

The patient was discharged without complications after surgery. The 3-year follow-up revealed that the patient recovered well with no evidence of recurrence.

**Lessons:**

Here, we emphasize the importance of careful radiological examination and reflect on the difficulty of tumor resection. Furthermore, understanding the treatment and diagnosis of lumbar paravertebral schwannoma is critical for plastic surgeons and radiologists when encountering similar cases.

## 1 Introduction

Schwannoma originating from the lumbar nerve is a rare and slowly growing tumor, which arises most commonly in the peripheral nervous system.^[1]^ The cause of schwannomas is still unknown, and its pathogenesis may be related to a lack of a mutation in *NF1*, which is associated with neurofibromatosis type I.^[2]^ Extraspinal schwannomas occur in the head, neck, upper and lower limbs, and posterior mediastinum. They typically present as single tumors, but occasionally present as multiple tumors. They rarely occur in the retroperitoneal spinal nerve roots; however, retroperitoneal tumors are the most common neurogenic tumors.^[3]^ The diagnosis and treatment of schwannomas in the lumbar spinal canal has been reported in a large number of studies, while giant paravertebral schwannomas near the lumbar nerve roots with bone destruction are reported less frequently and can be easily misdiagnosed or missed entirely.

The clinical symptoms of lumbar paravertebral schwannoma are usually nonspecific and generally develop late in the progression of the disease.^[4]^ Most patients are diagnosed when they develop a faint pain in the lower back or on physical examination. When the tumor compresses the nerve, it can manifest as lower back pain in the area of the diseased nerve root and radiation pain or soreness in the lower extremities.^[5]^ The symptoms gradually increase and the pain is not relieved after rest. Initially, there are no sensory disturbances or only regional hyperalgesia.^[4]^ When the spinal nerve root is compressed or strained, it can produce pain, further causing weakening of muscle strength and abnormal tendon reflex.^[6]^ In the current case study, the patient was diagnosed with a benign paravertebral schwannoma using the results of a magnetic resonance imaging (MRI) scan and a pathological examination rather than by the characteristic symptoms. Schwannomas can cause bone destruction, which is mainly caused by the compression of adjacent vertebral bodies and the expansion of intervertebral foramen. However, bone destruction caused by schwannomas is rarely reported.^[7]^

In this study, we describe a rare case of giant paravertebral schwannoma near the lumbar nerve roots with bone destruction in a 47-year-old woman.

## 2 Case report

A 47-year-old woman presented with only lower back pain and symptoms of bulging lumbar disc for 3 years, accompanied by limited movement and numbness. Referred for further investigation by the orthopedics department, a large spinal tumor was detected by MRI scan. She did not have any history of medical conditions, familial genetic conditions, food or drug allergies, or tuberculosis. In addition, the patient lost about 3 kg in body weight over the previous month. A further physical examination revealed no palpable head, neck, supraclavicular, axillary, or epitrochlear lymph nodes. A specialist examination revealed that the patient had a physiological curvature of the spine, and the activity was normal. There was no tenderness or searing pain in the lumbar spine or paravertebral spine, and there was no significant limitation on lumbar motion. Muscle strength and muscle tension were normal, muscles did not atrophy, physiological reflexes existed, and pathological reflexes were not elicited.

**Figure 1:**
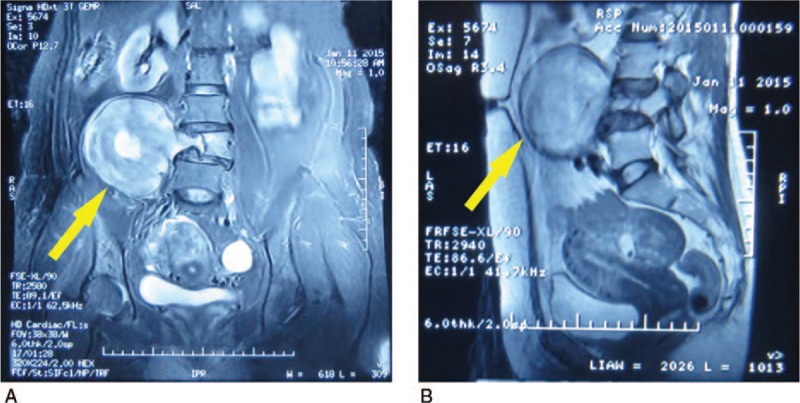
Preoperative magnetic resonance imaging (MRI) scan findings. A screening MRI showed a giant mass with uneven density originating from the 4th lumbar with bony destruction, and apparently there are several low-density areas within the tumor (A). The mass closely behind the psoas major (B).

Routine laboratory testing revealed normal levels of tumor markers such as carcinoembryonic antigen (CEA), alpha-fetoprotein (AFP), carbohydrate antigen 19-9, carbohydrate antigen 12-5, and carbohydrate antigen 153, but an elevated level of neuron-specific enolase (21.03 ng/mL, normal: 0–16.3 ng/mL). X-ray and 3-dimensional computed tomography scans showed a large mass on the right side of the lumbar 4 vertebral body, the lumbar intervertebral foramen 4 to 5 was dilated significantly, and the adjacent vertebral bodies and zygopophysis of the lumbar 4 were resorbed due to compression of the tumor. In addition, MRI showed a large mass with uneven density, 17 × 12 × 15 cm in size, located to the right of the 4th lumbar with obvious bony destruction (Fig. 1A) and closely behind the psoas major (Fig. 1B).

The operation was completed by orthopedic surgeons. We chose to operate via a combined anterior and posterior approach. First, a midline incision was made on the back of the waist with the lumbar 4 to the lumbar 5 spine as the center. After the separation of the anadesma and muscle beside the cone, bilateral facet joints and transverse processes of lumbar 2, 3, 5 and sacral 1 were exposed, and both sides were fixed with pedicle screws. Follow the right L4 lamina and the transverse decompression, we found her 4th lumbar vertebra was atrophic due to bone destruction by space-occupying lesions near the right nerve root of the lumbar spine. It appeared grayish-white, elastic, hard, and smooth, with a size of about 17 × 12 × 15 cm. The schwannoma was then separated from the nerve roots, and the tumor was partially removed and separated. Subsequently, the lower back fascia, subcutaneous tissue, and skin were sutured layer-by-layer. Next, we chose to operate via an anterior approach. Residual tumor was excised behind the psoas major completely through a lengthened incision at McBurney point. Furthermore, we took a tissue sample to perform a pathological examination. The surgeon took notice of the margin of the lesions without invasion into surrounding tissue, and confirmed that the tumor was benign. The operative time was 4 hours, and the estimated blood loss was 3000 mL. The patient needed a blood transfusion of 8 U of red blood cell suspension and 750 mL of fresh frozen plasma during the operation. There was no intraspinal involvement pre-op and intra-op. The patient had a normal operative course and recovered completely after 10 days.

**Figure 2:**
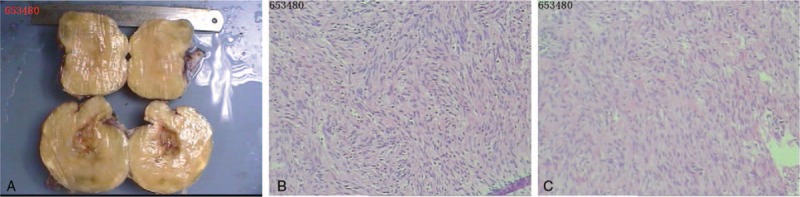
Gross findings. It appeared grayish-white, elastic, hard, and smooth, with a size of about 17 × 12 × 15 cm (A). Histological findings. Sectional cells exhibited large nuclei, strong staining, and no mitotic figures. In addition, tumor cells were diffusely positive for expression of S100 protein and both CD34 and desmin were partially negative (B and C).

**Figure 3:**
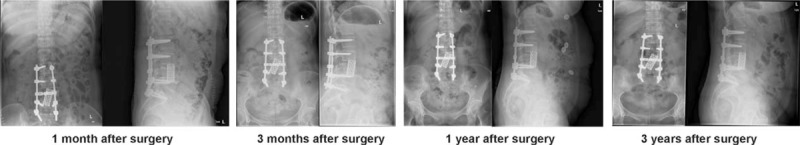
Follow-up data showed no evidence of tumor recurrence.

Inspecting the surgical sample by means of light microscopy revealed that the tumor was a benign schwannoma. The tumor was apparently spherical and circumscribed and rich in blood vessels. The cut surface was light-yellow with a hard texture. Histologically, the tumor consisted of a small number of atypical and fibroblast-like cells with a well-defined border. Some cells had a scattered arrangement, and cells with oval vacuolated cell nuclei were observed. Sectional cells exhibited large nuclei, strong staining, and no mitotic figures (Fig. 2A). Rarely, tumor cells were arranged into an interlaced and circinate shape, without a clear Verocay body. Immunohistochemical examination revealed that the tumor cells were diffusely positive for S100 protein expression (Fig. 2B) and locally negative for both CD34 (Fig. 2C) and desmin (Fig. 2D) expression. Based on these characteristics, we ultimately diagnosed the patient with a giant schwannoma originating from the lumbar. The schwannoma seemed to be successfully treated because the patient had no recurrence and no bony invasion during 3 years of follow-up (Fig. 3).

## 3 Discussion

Schwannomas are benign neoplasms arising from the nerve sheath. Occasionally, they are reported in locations such as the head, neck, peripheral nervous system, liver, pancreas, esophagus, stomach, and peritoneum.^[8–13]^ Lumbar paravertebral schwannomas with bone destruction near the lumbar nerve roots are rare and account for only 1% of schwannoma cases.^[14]^ Although the tumors arise from the peripheral nerve sheath, they rarely elicit any clinically detectable neurological deficits.^[15]^ Lumbar paravertebral schwannomas have no specific symptoms in the early stage and are difficult to diagnose. This type of schwannoma is easy to misdiagnose or miss entirely. We believe that there are 3 main reasons that lumbar paravertebral schwannomas are misdiagnosed: lumbar paravertebral schwannomas are rare and clinical manifestations are nonspecific; no specific tumor markers have been found that are indicative of the disease (eg, cancer antigen [CA]199, CEA, AFP, CA125, CA153); and tumor growth is slow, asymptomatic during the early phase, the retroperitoneal space of the tumor growth site is large, and the location is deep, so it is difficult to detect by physical examination. In this case, the schwannoma was detected late in the progression of the disease, with only the symptoms caused by lower disc bulge. There are case reports where schwannomas have been misdiagnosed as psoas abscesses^[16]^ and ovarian dermoid cysts^[17]^ at all ages. Tumor markers including AFP and CA125 contributed to the differential diagnosis of gynecological tumors versus schwannomas. However, our patient's tumor marker test (CA199, CEA, AFP, CA125, CA153) was negative. Therefore, a schwannoma should be considered when a patient tests negative for these markers. It is reported^[18]^ that 7.5% of gynecological tumors were positive for expression of AFP and 22.6% were positive for CA125. Hence, AFP and CA125 should not be the specific criteria for diagnosing gynecological tumors. Kim et al^[19]^ reported that strong immunohistochemical S100 protein staining was associated with schwannomas. Our patient's pathological examination of S100 was strongly positive in this study presentation and was also reported in the literature.

On the treatment side of schwannoma, radiotherapy and chemotherapy are not effective for this common and benign tumor.^[20]^ Currently, complete surgical resection of the tumor tissue is the best method of treatment. Schwannomas have a complete envelope, which is not tightly adhered to surrounding tissues such as soft tissues, spinal cords, and nerve roots. Therefore, they are easy to separate and can be completely removed. After complete resection of schwannomas, recurrence is rare, and only patients treated with partial resection have recurrence.^[21]^ However, we should note that the spine is rich with blood vessels, which favors hemorrhage during the operation. Tang et al^[22]^ reported that 39.88% of patients undergoing sacral tumor resection had blood loss greater than 3000 mL. Their data indicated that blood volume loss was influenced mainly by location, volume, and the blood supply of the tumor. Hemorrhage is a serious intraoperative risk in cases where major vessels are situated near the tumor, and there are currently several reports of unsuccessful tumor excisions and intraoperative mortalities.^[23]^ In this patient, the anterior and posterior combined laminectomy fully protected the facet joints. The advantage was that it could be exposed to decompression and had little effect on the stability of the spine. Due to the large schwannoma, after complete removal of the tumor and surrounding structures, the patient underwent bone fusion and internal fixation to achieve stable reconstruction of the spine and achieve proper healing. In short, to determine the best treatment for giant lumbar paravertebral schwannomas the following should be taken into account to reduce the incidence of surgical complications: the actual growth shape and size of the tumor, extent of invasion, relationship of the tumor with the dura mater, if the patient is a candidate for blood transfusions, surgical consultations, and classification to determine the best operation methods and protocols. The purpose of an operation is to remove the tumor as completely as possible, relieve pressure, and restore the function of the nerve and the spinal cord to the greatest extent, so as to maximize the success rate and prognosis of the operation.

In summary, we described a rare case of a giant paravertebral schwannoma near the 4th lumbar nerve roots with bone destruction. The most interesting finding is that the only clinical manifestation was a sensation of a bulging disc in the lower back. Although MRI cannot accurately provide a preoperative diagnosis, it can intuitively reflect the location and size of the tumor and the relationship with the surrounding organs and blood vessels. As for the tissue source of the tumor and distinguishing between benign and malignant, the diagnosis depends on surgical and pathological examinations. Moreover, we believe that extensive resection is useful for treating giant schwannomas and minimizing the risk of local recurrence, as our patient was free from disease at the 3-year follow-up.

## Author contributions

**Data curation:** Dai Min, Bin Zhang.

**Funding acquisition:** Dai Min, Bin Zhang.

**Methodology:** Ping Zhan, Yuan Liu.

**Project administration:** Hangjun Chen.

**Resources:** Ping Zhan, Yuan Liu.

**Supervision:** Bin Zhang.

**Writing – original draft:** Hangjun Chen.

**Writing – review & editing:** Qiang Xu.
